# Influence of cultural capital and economic level on the learning of Mexican students

**DOI:** 10.3389/fpsyg.2026.1681122

**Published:** 2026-03-02

**Authors:** Eduardo Hernández-Padilla, Aldo Bazán-Ramírez, Danivia López-García, Edwin Félix-Benites, Wilfredo Bazán-Ramírez, Edgar Gutiérrez-Gómez

**Affiliations:** 1 Center for Transdisciplinary Research in Psychology, Universidad Autónoma del Estado de Morelos, Cuernavaca, Mexico; 2 School of Psychology, Universidad Nacional José María Arguedas, Andahuaylas, Peru; 3 School of Higher Studies Mazatepec, Universidad Autónoma del Estado de Morelos, Cuernavaca, Mexico; 4 Faculty of Educational Sciences, Universidad Nacional del Callao, Callao, Peru; 5 Faculty of Administrative Sciences, Universidad Nacional Mayor de San Marcos, Lima, Peru; 6 Universidad Nacional Autónoma de Huanta, Ayacucho, Peru, Ayacucho, Peru

**Keywords:** academic achievement, basic education, cultural capital, economic level, language

## Abstract

**Introduction:**

The influence of the family’s socioeconomic and cultural level has been one of the most analyzed factors in student achievement. While cultural capital and economic level are linked, previous research suggests they exert differential effects on learning. This study aimed to compare the influence of these two factors on the language and communication achievement of Mexican elementary and middle school students in the Planea test.

**Methods:**

Structural equation modeling (SEM) was employed to analyze the data through path analysis, allowing for a precise comparison of the variables across different educational levels.

**Results:**

The findings reveal significant differences between the influence of cultural and economic capital. These effects are associated with the type of school support and the specific educational level of the students.

**Discussion:**

In agreement with previous studies, the results suggest the importance of evaluating these factors separately. The magnitude of influence for each factor shifts throughout different educational stages, highlighting the need for nuanced academic achievement assessments.

## 1 Introduction

Since the classic Coleman Report (Coleman et al., 1966), family socioeconomic status has been identified as an important source of influence on student academic achievement, emerging as a key factor in explaining differences in achievement gaps among students of diverse backgrounds. The relationship between cultural capital and educational outcomes has been extensively documented in classical and contemporary literature (Bourdieu, 2019; Carabaña, 2016), while recent studies have further explored how family background and social heritage influence academic trajectories under diverse structural conditions (Breinholt and Jaeger, 2020; Jaeger and Karlson, 2018; Kosunen et al., 2021; Sieben and Lechner, 2019). These unequal and inequitable outcomes are critical to understanding the persistent gaps in student achievement across different educational systems (Guhn et al., 2020), leading to a body of research demonstrating that socioeconomic and family-of-origin factors strongly determine academic success (Bazán et al., 2022; Hee and Shuhan, 2022; Liu and Liao, 2023; Pishghadam et al., 2023; Vargas-Sánchez, 2023).

Throughout different international assessments implemented by the OECD (Organization for Economic Cooperation and Development) with the PISA (Programme for International Student Assessment) test (OECD, 2016; OECD - Organisation for Economic Cooperation and Development, 2019; OECD - Organisation for Economic Cooperation and Development, 2023); and those carried out by the Latin American Laboratory for the Evaluation of the Quality of Education (LLECE) with the Regional Comparative and Explanatory Study (UNESCO – United Nations Educational, Scientific and Cultural Organization, 2021; UNESCO, 2016; UNESCO, 2013), organized by UNESCO (United Nations Educational, Scientific and Cultural Organization), have consistently shown that the socioeconomic and cultural level of students is positively associated with the level of achievement and learning in standardized tests.

In the case of Mexico, during its participations in international assessments, it has shown results like the rest of the countries, students with a higher socioeconomic level obtain the best achievement results, while the least favored students have the results with the lowest values (Instituto Nacional para la Evaluación de la Educación – INEE, 2016). In the case of national assessments, in the EXCALE (Examinations of Educational Quality and Achievement) and Planea (National Plan for the Evaluation of Learning) tests, the results are similar (Instituto Nacional para la Evaluación de la Educación – INEE, 2019a, 2019b, 2017).

In the Mexican context, these findings have generated countless studies aimed at assessing the effect of cultural capital and socioeconomic level of origin on educational achievement. Despite progress in improving the quality of basic education, the Mexican educational landscape continues to show wide educational gaps strongly associated with students’ socioeconomic and cultural backgrounds (Backhoff-Escudero, 2018; Blanco, 2023; Bazán-Ramírez et al., 2016; Hernández-Padilla and Bazán, 2016; Moreles-Vázquez, 2024; Prieto et al., 2013). To contextualize these variables, we considered the standards established by national and international evaluation bodies (Asociación Mexicana de Agencias de Inteligencia de Mercado y Opinión, 2023; Instituto Nacional para la Evaluación de la Educación – INEE, 2018), which provide a robust basis for analyzing inequality and justify the selection of indicators in this research (Córdoba Perozo, 2016; Downey and Condron, 2016). This emphasis on joint influence often overlooks significant differences between specific cultural capital factors—such as parental education and educational expectations—and household economic wealth, a gap this study aims to address (Backhoff-Escudero, 2018; Bazán et al., 2016, 2022; Blanco-Bosco, 2023; Hernández-Padilla and Bazán, 20116; Hernandez-Padilla et al., 2023; Moreles-Vázquez, 2024; Prieto et al., 2013; Vargas-Sánchez, 2023).

In contrast, some studies have analyzed separately the effects that economic level (such as the possession of various goods and access to certain types of services) and cultural capital (such as parents’ schooling, the number of books at home, attendance at cultural events, etc.) have on the learning and/or academic achievement of students, mainly in elementary, middle and high school. In these works, significant differences have been observed between the influence that family cultural and economic capital have on learning or academic achievement (Zhu, 2023; Xie and Ma, 2019; Hernández-Padilla, 2011). Because of this differential analysis, some studies have shown that socioeconomic status is more important than Cultural Capital on academic achievement (Lehti and Kinnari, 2024; Thomsen, 2021; Zarifa et al., 2018; Klausen, 2016); meanwhile, other authors indicate that cultural capital is more strongly linked to students’ learning or educational achievement (Xie and Hutagalung, 2025; Pishghadam and Zabihi, 2011).

A main characteristic of works analyzing cultural capital is the diversity of indicators used to represent it, due to the lack of a consensually accepted operational definition. In addition to the above, the selection of appropriate indicators to represent cultural capital becomes more difficult according to the theoretical definition of capital (Bourdieu and Jiménez, 2011; Bourdieu and Passeron, 1977). According to the aforementioned authors, three forms of capital are considered: the objectified cultural capital, which refers to educational resources at home (reading materials, learning aids, etc.) and cultural resources (books of classical literature, works of art, etc.); the incorporated cultural capital, which refers to the resources of the home (books of classical literature, works of art, etc.); the embodied cultural capital, which refers to the resources of the home (books of classical literature, works of art, etc.) and cultural resources (books of classical literature, works of art, etc.); the embodied, which refers to most of the family characteristics of the student body, and which occurs mainly in families where socioeconomic status is linked to educational achievement (such as attitudes and values prone to learning; tastes and preferences directed toward academic goals, and the influence of mastery and aptitude of academic skills); and finally, the institutionalized capital, which is a publicly recognized social distinction of what is in turn institutionalized cultural capital (Bourdieu and Jiménez, 2011; Bourdieu and Passeron, 1977).

Tan (2017) points out in his meta-analysis that the study of cultural capital is not a unidimensional construct with consistent effects on achievement, but can manifest itself in different ways, with some variables showing a stronger association than others. Indeed, the results reported by Tan (2017) show that institutionalized cultural capital (represented by parental education) has a more significant effect than embodied cultural capital. Not surprisingly, parental education has been employed in many previous studies to measure institutionalized cultural capital on educational attainment (Ko et al., 2024; Kallunki and Purhonen, 2017; Klausen, 2016; Lehti and Kinnari, 2024; Willekens et al., 2014).

In the case of large-scale international assessments such as PISA and ERCE, and at the national level such as Planea in Mexico, several research have used as indicators of cultural capital the schooling of parents, and the number of existing books at home, where cultural capital has been shown to have a significant impact on academic achievement, and this influence is of a positive type, that is, as cultural capital grows the results in achievement also increases (Backhoff-Escudero, 2018; Blanco-Bosco, 2023; Bazán-Ramírez et al., 2016, 2022; Hernández-Padilla, 2016; Moreles-Vázquez, 2024; Prieto et al., 2013).

The objective of the present study was to compare the effects of economic level and cultural capital (mainly in its institutionalized form) on the achievement of Mexican students in Language and Communication in the Planea 06 and 09 tests (sixth grade and third grade, respectively).

## 2 Method

### 2.1 Design and participants

The present study presents an ex-post-facto or non-experimental design, since there is no experimental control over the variables considered for analysis.

The database of the PLANEA 09 test (school year 2016–2017) was used for the present study, in which 131,662 third-year high school students participated, 50.4% were female. The average age was 15.17 years, with a standard deviation of 0.58. The sample came from 3,358 schools, 85.1% of which were public schools. For its part, the PLANEA 06 test (2017–2018 school cycle), in which 104,973 participated (50.1% female, average age 12.03 and a standard deviation of 0.54), coming from 3,491 schools, 89.6% being public. Table 1 shows the disaggregated information.

**Table 1:** Characteristics of the samples studied in the present study for the Planea 06 test (2017–2018 school year) and Planea 09 (2016–2017 school year).

Test	Type of service	Sample size (n)		Age		Sex percentage of females
		Schools	Students	Mean	S. D.	
Planea 06	Public	3,128	92,948	12.03	0.51	50.3
	Private	363	12,025	12.04	0.46	48.9
	Total	3,491	104,973	12.03	0.51	50.1
Planea 09	Public	2,856	110,329	14.68	0.67	50.2
	Private	502	21,333	14.68	0.58	51.3
	Total	3,358	131,662	14.68	0.65	50.4

Source: Own elaboration based on student context questionnaires Planea 06 (2017–2018 school cycle) and Planea (2016–2017).

### 2.2 Variables

For the construction of the Cultural Capital and Economic Level factors, three and 18 common variables were used, in that order, extracted from the context questionnaire of students in sixth grade of primary and third grade of secondary school. For the Cultural Capital factor, indicators of the educational level of the father and mother were used, as well as the number of books at home (excluding textbooks provided by the school). On the other hand, for Economic level we considered the characteristics of the house (number of light bulbs and type of floor); the goods owned (washing machine, refrigerator, stove, microwave oven, fixed telephone, computer or laptop, etc.) as well as services (electricity, drinking water, drainage, pay TV, and Internet access). Table 2 shows the factors and their corresponding indicators, and the internal consistency analysis (Ordinal Alpha) of the former. It is possible to appreciate in said table that the level of consistency for the Cultural Capital factor is unacceptable (0.55 for Planea 06 and 0.57 in Planea 09; Reidl-Martínez, 2013), this may be attributable to the number of indicators that are employed. In contrast, the consistency obtained for Economic level is good for both tests (0.77 and 0.81, for Planea 06 and Planea 09, in that order).

**Table 2:** Indicators used in the construction of the cultural capital and economic level factors; the ordinal alpha coefficients are shown.

Indicators	Planea 06	Planea 09
Capital of culture		
Father’s educational level	0.55	0.57
Mother’s educational level		
Number of books at home		
Economic level		
Number of light bulbs in house	0.77	0.81
Services: Electricity		
Services: Drinking water		
Services: Drainage		
Services: Solid floor		
Appliances: Washing machine		
Appliances: Refrigerator		
Appliances: Microwave oven		
Appliances: Gas stove or electric stove		
Appliances: Landline telephone		
Amenities: Pay TV (Sky, Dish, Netflix, cable TV, etc.)		
Services: Internet access		
Assets: Computer or laptop		
Goods: Television or flat screen		
Assets: Car or truck		
Goods: Mobile phone or cell phone		
Goods: Tablet (electronic tablet)		
Goods: DVD, Blu-ray (video disk player)		

Source: Own elaboration based on student context questionnaires Planea 06 (2017–2018 school cycle) and Planea (2016–2017).

Both factors were constructed through an exploratory factor analysis, using principal component extraction (restricted to the extraction of a single factor), and employing Varimax rotation; scores were created for each student using the Regression option with a mean of zero and a standard deviation of one. Table 3 shows the descriptive values of the factors by test and type of school.

**Table 3:** Descriptive values of the cultural capital and economic level factors by test and type of school.

Type of school	Factor	N	Minimum	Maximum	Mean	S. D.
Planea 06						
General	Family Capital Cultural	26,080	−2.13	1.74	0.00	0.96
	Economic level of the family	26,080	−4.88	2.52	−0.01	0.94
Public	Family Capital Cultural	23,183	−2.13	1.74	−0.07	0.91
	Economic level of the family	23,183	−4.88	2.52	−0.12	0.90
Private	Family Capital Cultural	2,897	−2.13	1.74	0.55	1.15
	Economic level of the family	2,897	−2.90	2.52	0.89	0.73
Planea 09						
General	Family Capital Cultural	131,662	−2.56	1.96	0.00	0.98
	Economic level of the family	131,662	−4.57	1.82	0.00	0.98
Public	Family Capital Cultural	110,329	−2.56	1.96	−0.16	0.90
	Economic level of the family	110,329	−4.57	1.82	−0.18	0.93
Private	Family Capital Cultural	21,333	−2.56	1.96	0.85	0.97
	Economic level of the family	21,333	−4.57	1.82	0.94	0.57

S. D., Standard Deviation. Source: Own elaboration based on student context questionnaires Planea 06 (2017–2018 school cycle) and Planea (2016–2017).

## 3 Procedure

### 3.1 Data analysis

Descriptive analyses of the variables used in the study of the Planea 06 and 09 tests were performed with the SPSS ver. 23 statistical program. The reliability analysis, the ordinal alpha, and the construction of the factors Cultural capital and Economic level were carried out with the software Factor Analysis (Lorenzo-Seva and Ferrando, 2022). Likewise, the EQS 6.4. program was used (Bentler, 1995), and goodness-of-fit criteria such as χ2; RMSEA (root mean squared residuals of approximation), with acceptable values between 0.08–0; CFI (comparative goodness-of-fit index), with acceptable interval of 0.09–1.0; and TLI (Tuker-Lewis index), also with interval between 0.9–1 (Mulaik et al., 1989; Hooper et al., 2008; Dragan and Topolšec, 2014).

**Figure 1:**
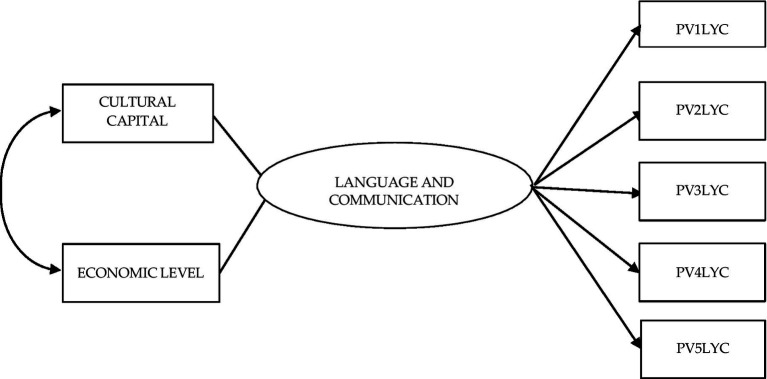
Structural equation model to evaluate the effect of the independent variables cultural capital and economic level on performance in language and communication (covariance between independent variables is established).

Figure 1 shows the theoretical model that, in the structural equations, was subjected to analysis. It shows the direct effects of the Cultural Capital and Economic Level factors, and the covariance between them, on the latent factor Language and communication and their respective five plausible observed values (Table 4).

**Table 4:** Descriptive values language and communication by test and type of school.

Application	Type of school	N	Minimum	Maximum	Mean*	S. D.
Planea 06 (school cycle 2017–2018)	General	104,973	174.26	882.29	507.62	98.14
	Públicas	92,948	174.26	850.18	495.42	91.75
	Public	12,025	290.77	882.29	601.96	94.67
Planea 09 (school cycle 2017–2018)	General	131,662	130.16	947.04	500.71	113.29
	Públicas	110,329	130.16	879.96	483.06	106.08
	Public	21,333	209.92	947.04	592.00	105.16

S. D., Standard Deviation. *Average from the five plausible values for Language and communication. Source: Own elaboration based on data from the context questionnaires of students participating in the Planea 06 and Planea 09 tests.

## 4 Results

To shorten the presentation of the results, in general terms, in the six different models carried out, the error terms associated with the manifest variables (the five plausible values of Language and Communication) had values of 0.89, while the error associated with the Language and Communication factor was 0.14. Regarding the values of the coefficients between the Language and Communication factor and each of its five different plausible values, the magnitude was 0.94 in the six models analyzed.

**Figure 2:**
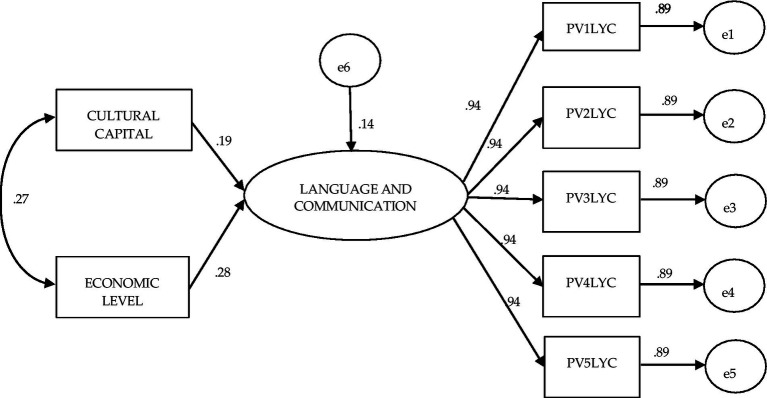
Structural equation model to evaluate the effect of the independent variables cultural capital and economic level on achievement in language and communication: Planea 06 general.

Figure 2 shows the structural equation model obtained for sixth grade students in all schools (Planea 06). The values of the goodness of fit criteria used show an excellent fit to the proposed model (χ^2^ (df = 13) = 5.226 *p* = 0.970; BBNFI = 1.00; CFI = 1.0; AGFI = 1.0; SRMR = 0.000; RMSEA = 0.000–interval 0.000–0.000). In this figure, we can see that the covariance between Cultural Capital and Economic Level is 0.27, while the influence of each of the different factors on language and communication is different: while Cultural Capital has a coefficient of 0.19, Economic Level is 0.28. Regarding the values of the coefficients between the factor Language and Communication and each of its five different plausible values, the magnitude was 0.94 in the six models analyzed.

**Figure 3:**
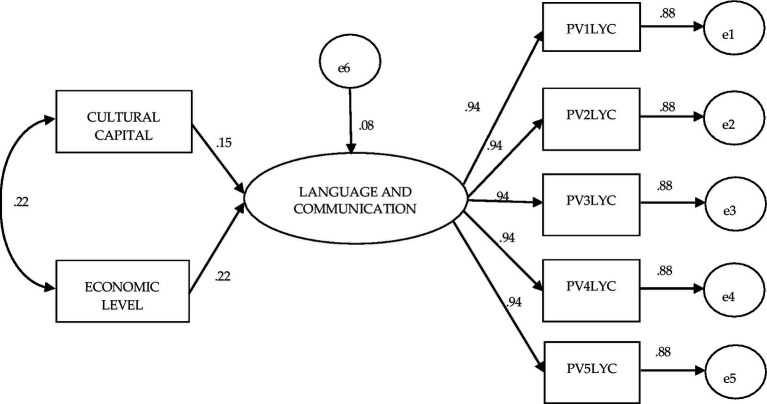
Structural equation model to evaluate the effect of the independent variables cultural capital and economic level on achievement in language and communication: Planea 06 public schools.

A similar trend on the effects of Cultural Capital and Economic Level on Language and communication learning was observed in sixth grade students from public schools, where the ratio of the above factors were 0.22; while their effects on Language and communication were 0.15 for Cultural Capital and 0.22 for Economic Level. The model showed excellent goodness-of-fit values (χ^2^ (df = 13) = 13.227 *p* = 0.450; BBNFI = 1.00; CFI = 1.0; AG-FI = 1.0; SRMR = 0.000; RMSEA = 0.001–range 0.001–0.001; see Figure 3).

**Figure 4:**
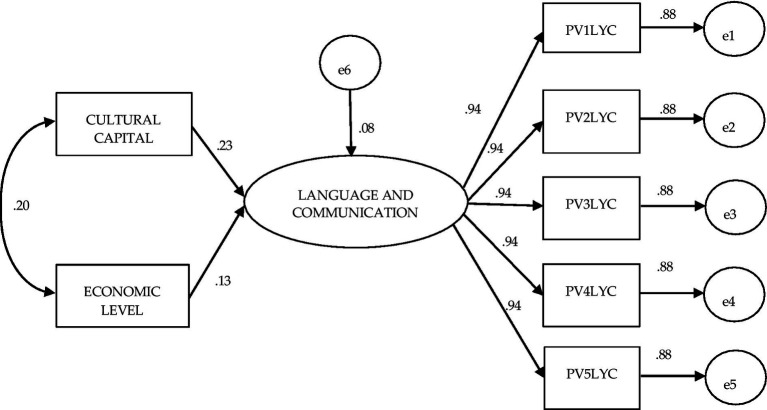
Structural equation model to evaluate the effect of the independent variables cultural capital and economic level on achievement in language and communication: Planea 06 private schools.

In contrast to what was observed in the general and public schools, Figure 4 shows the results of the modeling based on the information of students from private schools: the influence of Cultural Capital is greater than that of Economic Level on learning in Language and Communication (0.23 and 0.13, respectively); and with a covariance between the factors of 0.20, lower than the previous covariances. This model, like the two previous models, also showed excellent value in the goodness-of-fit criteria (χ^2^ (df = 13) = 4.368 *p* = 0.987; BBNFI = 1.00; CFI = 1.0; AGFI = 1.0; SRMR = 0.000; RMSEA = 0.000–interval 0.000–0.000; see Figure 4).

**Figure 5:**
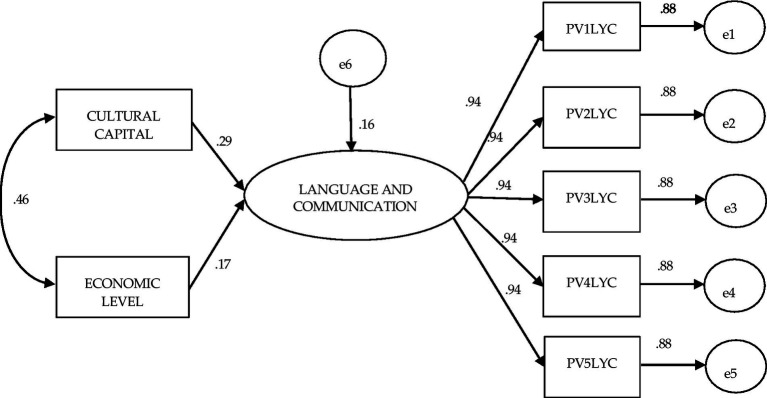
Structural equation model to evaluate the effect of the independent variables cultural capital and economic level on achievement in language and communication: Planea 09 General.

Figure 5 presents the structural equation modeling for all third-year high school students who participated in Planea 09. As in the previous models, very good fit values were obtained in this model (χ^2^ (df = 13) = 4.368 *p* = 0.987; BBNFI = 1.00; CFI = 1.0; AGFI = 1.0; SRMR = 0.000; RMSEA = 0.000—interval 0.000–0.000). In contrast to what was observed in the general Planea 06 model, in this model the influence of Cultural capital on Language and communication is greater than that of Economic level (0.29 and 0.17, respectively). In the same sense, the covariance between both factors is greater (0.46), indicating a greater association between factors than in the previous models. This model shows that the influence of Cultural Capital begins to have more value than Economic Level in secondary education, as can be seen in the subsequent models.

**Figure 6:**
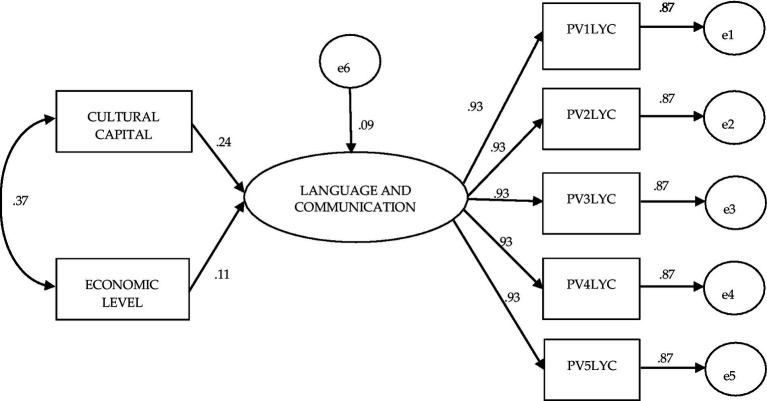
Structural equation model to evaluate the effect of the independent variables cultural capital and economic level on achievement in language and communication: Planea 09 public.

The growing importance of Cultural Capital in student learning, compared to Economic Level, can be seen in Figure 6, which shows the model for students from public schools in the third year of secondary school. The influence of Cultural Capital (0.24) is greater than that of Economic Level (0.11) on Language and Communication learning, while the association between both factors is also greater than that of the previous model, from public elementary schools (0.37). This model also had good fit values for structural equation modeling: χ^2^ (df = 13) = 8.455 *p* = 0.813; BBNFI = 1.00; CFI = 1.0; AGFI = 1.0; SRMR = 0.000; RMSEA = 0.000–range 0.000–0.002.

**Figure 7:**
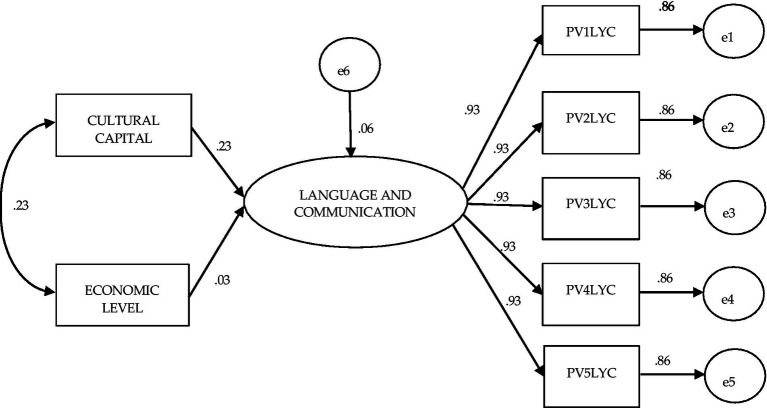
Structural equation model to evaluate the effect of the independent variables cultural capital and economic level on achievement in private language and communication: Planea 09.

Finally, the sixth model, shown in Figure 7, corresponds to private secondary schools; the influence of Economic Level, although positive, is marginal (only 0.03 points), while the only significant influence is that of Cultural Capital (0.23) on achievement in Language and Communication. On the other hand, the covariance between both predictor variables decreases with respect to the previous model (0.23). Concerning the model fit values, it also obtained good modeling results (χ^2^ (df = 13) = 7.821 *p* = 0.855; BBNFI = 0.99; CFI = 1.0; AGFI = 1.0; SRMR = 0.000; RMSEA = 0.000–range 0.000–0.004).

## 5 Discussion

In this paper, we analyzed the differential effects that Cultural Capital and Economic Level had on the Language and Communication achievement of Mexican students in sixth grade of primary and third grade of secondary school. For this purpose, a theoretical model was proposed that resulted in six different types (one for each educational level, and two for type of support, public and private, at each level). Our findings align with recent global evidence regarding the influence of socioeconomic status on learning outcomes (Adedeji et al., 2023; Tan and Fang, 2023). Furthermore, the application of advanced modeling techniques, such as the structural equations (SEM) used in this study, aligns with modern methodological trends and rigorous validation standards in social sciences (Assulaimani and Althubaiti, 2021; Imbulana-Arachchi and Managi, 2023; Jin et al., 2022, 2024; Marsman et al., 2016; Mikus et al., 2020). Consequently, the six different resulting models obtained good goodness-of-fit criteria according to the specialized literature (Dragan and Topolšek, 2014; Hooper et al., 2008; Mulaik et al., 1989).

The modeling results show differential effects of the student’s cultural capital and economic level, not only between the two educational levels, but also in the type of school support. The growing importance that cultural capital has on academic achievement, in contrast to the wealth of the students’ family (the latter made up of resources, possessions and services in the home), can be seen in the results of the modeling carried out with the data of the students of both educational levels, the difference in the effects of economic lev-el in the models for sixth and third year of high school; these differential results by educational levels have been pointed out by various authors (Zhao, 2024; Yang, 2023; Yu et al., 2022). The greater influence that Cultural Capital has than Economic Level; may be attributable to the direct benefits of the cultural resources available in the home (for example, the number of books at home; Blanco-Bosco, 2017), having parents with a high educational level (Espejel-García and Jiménez-García, 2019), and, consequently, having a greater institutionalized capital (Bourdieu and Jiménez, 2011; Bourdieu and Passeron, 1977), with the benefits that this entails.

In contrast, it is possible to observe that the economic level is important in the first educational levels, mainly in public schools where, given the educational inequalities resulting from the educational system, household resources play a very important role (Blanco-Bosco, 2023, 2017; Blanco-Bosco, 2011); while in private schools the school environment, the school context, is one of the most important variables associated with educational achievement/learning (Bazán-Ramírez et al., 2022; Bazán-Ramírez et al., 2016; Hernández-Padilla and Bazán-Ramírez, 2016). In a broader sense, students from schools made up of students with a very low socioeconomic level can only be favored if they are placed in schools with a better school context than those of their origin (Graña and Murillo, 2023; Murillo et al., 2023; Murillo, 2016).

For their part, other authors suggest that it is very important that parents or legal guardians of students promote greater knowledge by the educational level of the family, encourage various activities that promote different types of family cultural capital, increase the student’s educational expectations, have greater parental participation in school and extracurricular activities, and increase to the extent of the family’s economic and social capabilities, the investment in cultural resources to which students can have access (Xie and Hutagalung, 2025; Zhu, 2023; Xie and Ma, 2019).

The results show that cultural capital has a stronger association with academic achievement than family economic level, particularly in later stages of compulsory education. This finding supports the need for school-based practices that promote access to culturally valued learning experiences, such as reading and academic language use, especially in public schools serving disadvantaged populations (Bourdieu, 1986; Blanco-Bosco, 2017). The greater relevance of economic level in early educational stages highlights the continued importance of compensatory policies ensuring minimum material and educational conditions in primary education, particularly in contexts characterized by structural inequalities (Blanco-Bosco, 2017).

In addition, the differential effects observed by school type indicate that improving school context through investments in instructional quality, school climate, and institutional stability—may help mitigate socioeconomic disadvantages (Murillo, 2016; Graña and Murillo, 2023). Finally, the results support family-oriented policies that encourage parental involvement, educational expectations, and access to cultural resources, reinforcing a multidimensional approach to educational equity (Xie and Hutagalung, 2025; Zhu, 2023).

The findings of the present study must be interpreted with caution, as it does not constitute longitudinal study. This is primarily attributable to the fact that the data under analysis originates from two distinct samples collected in different years. Despite their representativeness at the national level, these data do not offer insight into the temporal evolution of cultural capital and economic level. Similarly, the absence of higher education data precludes the ability to ascertain whether these trends in both factors are maintained at higher educational levels than those observed. The rationale behind the exclusion of high school data from the analysis is attributable to the fact that the context questionnaires of the Planea 12 test of the year did not include the same indicators of economic level as those included in this research.

Among the main limitations of the work is the representation in terms of indicators of the factors analyzed, as well as the ages analyzed. In the first instance, there are serious difficulties in the choice of the most appropriate indicators to represent cultural capital, as well as its influence on students learning and/or achievement, since it does not have a unidimensional definition, and that some variables used for its construction have a greater effect than others. While the obtained Cronbach’s alpha (0.55) falls short of the conventional 0.70 benchmark, this coefficient is defensible given the scale’s reliance on only three indicators. In accordance with Briggs and Cheek (1986), internal consistency in parsimonious scales is more accurately validated through inter-item correlation rather than Alpha coefficients alone. From a theoretical standpoint, the multifaceted nature of Cultural Capital (Bourdieu, 1986) explains why institutionalized state indicators (parental education) and objectified state indicators (books at home) exhibit theoretical complementarity rather than linear redundancy (Sullivan, 2001). Furthermore, as a sociodemographic proxy, this construct inherently encompasses a broad multidimensionality (Giraldo and Vásquez, 2019), which naturally limits internal homogeneity in favor of a more comprehensive structural measurement. However, it is important to note that the indicators used in this study for shaping the Cultural Capital factor (parents’ educational level, specifically), have been consistently employed in representing it and the effects it has on students’ learning and/or achievement in various studies (Xie and Hutagalung, 2025; Tan, 2017).

## 6 Conclusion

The present study delved into the complex relationship between cultural capital and the economic level of Mexican students, analyzing its impact on performance in Language and Communication in sixth grade of primary school and third grade of secondary school. The results of the proposed models, which showed a good fit, reveal differential effects of these variables, not only between educational levels but also between public and private schools. The analysis underscores the growing influence of cultural capital on academic performance, in contrast to the family economic level. This finding is consistent with the literature that highlights the importance of cultural resources at home, such as the number of books, and the educational level of parents, which translates into greater institutionalized capital (Bourdieu and Jiménez, 2011; Bourdieu and Passeron, 1977). These factors seem to have a more pronounced effect as students’ progress in their educational trajectory.

On the other hand, it was observed that economic level plays a more relevant role in the early educational stages, especially in public schools. This suggests that, given the inherent inequalities in the education system, household material resources act as a crucial compensatory factor (Blanco-Bosco, 2023; Blanco-Bosco, 2017; Blanco-Bosco, 2011). In contrast, in private schools, the school context emerges as a more determining variable for academic achievement (Bazán-Ramírez et al., 2022; Hernández-Padilla and Bazán-Ramírez, 2016). The evidence suggests that improving the school context can mitigate the disadvantages of low-income students (Graña and Murillo, 2023; Murillo, 2016). The findings of this study highlight the need for educational policies to focus on strengthening the cultural capital of families. Authors such as Xie and Hutagalung (2025) and Zhu (2023) have suggested strategies for parents to promote cultural activities, increase educational expectations and school participation, and invest in cultural resources. However, it is essential to interpret these results with caution.

The nature of the study, which is not longitudinal, prevents the analysis of the temporal evolution of these variables. Furthermore, the absence of data from upper secondary education limits the ability to generalize these trends to higher education levels. The limitations of the study include the difficulty in representing cultural capital through unidimensional indicators, although validated variables were used from the literature.

A key limitation of this study relates to the operationalization of cultural capital using data from the PLANEA contextual questionnaires. Consistent with large-scale educational research, cultural capital was measured through parental educational attainment and the number of books at home, representing the institutionalized and objectified forms of the construct (Bourdieu, 1986; Sullivan, 2001). While these indicators show robust associations with academic achievement, particularly in language-related domains (Xie and Hutagalung, 2025; Blanco-Bosco, 2017; Tan, 2017), they capture only a partial dimension of cultural capital.

Specifically, this approach does not account for embodied forms of cultural capital, such as habitus, parenting practices, or everyday cultural dispositions, which have been shown to shape students’ academic trajectories (Lareau and Weininger, 2003; Kingston, 2001). This limitation reflects constraints of the available data rather than a theoretical omission. Future research incorporating broader measures and longitudinal designs would allow for a more comprehensive assessment of how different forms of cultural capital interact across educational stages. For future research, it is recommended to develop longitudinal studies that allow tracking the impact of these variables over time and exploring how they manifest at different educational stages.

## Data Availability

The original contributions presented in the study are included in the article/supplementary material, further inquiries can be directed to the corresponding authors.
